# 2303. Detection of COVID-19 Outbreaks in Hospitals Using Built Environment Testing for SARS-CoV-2

**DOI:** 10.1093/ofid/ofad500.1925

**Published:** 2023-11-27

**Authors:** Lucas Castellani, Derek MacFadden, Jason Moggridge, Bryan Feenstra, Makenna Wiebe, Sawith Abey, Evgueni Doukhanine, Michael Fralick, Aaron Hinz, Laura Hug, Nisha Thampi, Alex Wong, Rees Kassen, Caroline Nott

**Affiliations:** Sault Area Hospital, Sault Ste Marie, Ontario, Canada; The Ottawa Hospital Research Institute, Ottawa, Ontario, Canada; Mt Sinai Hospital, Toronto, Ontario, Canada; The Ottawa Hospital, Ottawa, Ontario, Canada, Ottawa, Ontario, Canada; Sault Ste Marie Academic Medical Association, Sault Ste Marie, Ontario, Canada; Sault Ste Marie Academic Medical Association, Sault Ste Marie, Ontario, Canada; DNA Genotek Incorporated, Ottawa, Ontario, Canada, Ottawa, Ontario, Canada; University of Toronto Department of Medicine, Toronto, Ontario, Canada; Carleton University, Ottawa, Ontario, Canada; University of Waterloo, Waterloo, Ontario, Canada, Waterloo, Ontario, Canada; CHEO, Ottawa, Ontario, Canada; Carleton University, Ottawa, Ontario, Canada; University of Ottawa, Ottawa, Ontario, Canada; The Ottawa Hospital, University of Ottawa, Ottawa Hospital Research Institute, Ottawa, Ontario, Canada

## Abstract

**Background:**

Environmental testing for SARS-CoV-2 including assessment of wastewater and the built environment is a useful tool for population-level surveillance for COVID-19. Detection of SARS-CoV-2 on the floor of healthcare facilities has been strongly associated with cases of COVID-19 (1,2). By introducing routine floor-swabbing in hospitals, we may be able to predict outbreaks earlier allowing for additional control measures.

**Methods:**

We implemented floor swabbing surveillance for SARS-CoV-2 to aid the identification of COVID-19 cases and outbreaks in hospitals. Swabs were taken weekly at eight hospital in-patient wards in healthcare worker-only (HCW) areas at two hospitals in Ontario, Canada, for a 39-week period (July 2022 to March 2023). HCW cases and outbreaks were managed as per usual processes at the facilities. A logistic regression model with ward-level random intercepts was developed using weekly viral copies (VC) to predict a contemporaneous outbreak in the same ward in the same week. Grouped 5-fold cross-validation was used to evaluate model outbreak discrimination.

**Results:**

SARS-CoV-2 RNA was detected on 537 of 760 collected swabs (71%). Hospital A had more frequent detection and higher levels of SARS-CoV-2 (swab positivity = 90% [95% CI: 85%-93%], mean VC = 23, [19-29]) than Hospital B (swab positivity = 60% [55%-64%], mean VC = 7.9 [6.5-9.7]) (Figure 1). There were seven outbreaks at Hospital A and four at Hospital B. Outbreaks at both hospitals consisted of mostly patient cases (Hospital A: 95%, Hospital B: 82%). The odds ratio of outbreak for every unit increase in viral copies (log-transformed) was 21.0 [5.6-79]. The cross-validated area under the receiver operating curve for SARS-CoV-2 viral copies for predicting a contemporaneous outbreak (Figure 2) was 0.86 [95%CI 0.82 – 0.9].

Figure 1.

**Figure:**
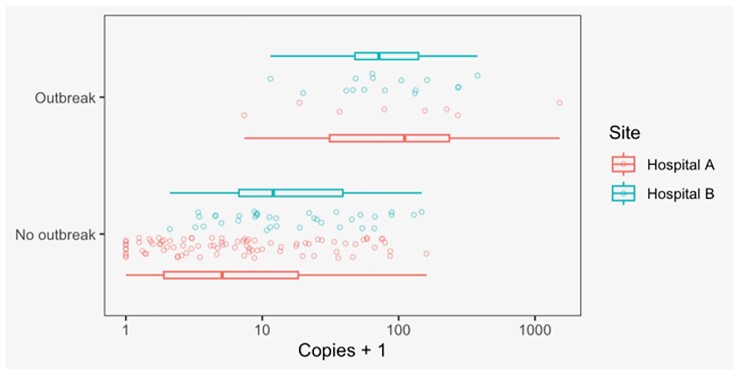

Distribution of SARS-CoV-2 copies (plus one) values from PCR testing of floor swabs, stratified by hospital and outbreak status at time of sampling. Jittered points show the copies plus one values for each individual swab; boxplots show the median, IQR, and range of these values by site (indicated by colors), in outbreak and non-outbreak periods (indicated on y-axis).

Figure 2.

**Figure:**
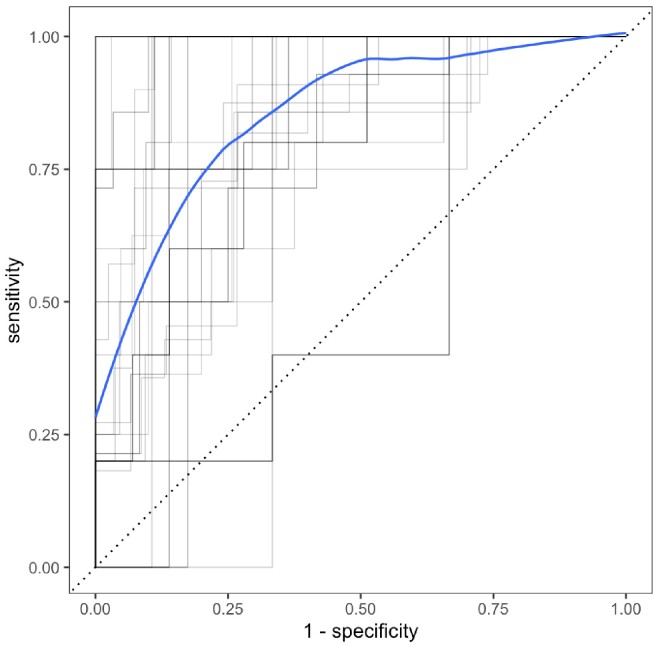

Cross-validation receiver operating characteristic (ROC) curves (with mean ROC in blue) for predicting contemporaneous outbreaks from SARS-CoV-2 viral copies.

**Conclusion:**

Detection of SARS-CoV-2 on floors in HCW-only areas is associated with COVID-19 outbreaks in those hospital wards. Despite swabbing exclusively in HCW-only areas, outbreaks were driven by patient cases at both hospitals. These results support the potential role for built environment sampling to support hospital COVID-19 outbreak identification and may fill gaps in traditional clinical surveillance methods.

**Disclosures:**

**Evgueni Doukhanine, MSc**, DNA Genotek: DNA Genotek provided sampling swabs in-kind for this study in an unrestricted fashion. **Michael Fralick, MD**, ProofDx: Advisor/Consultant

